# Health of Cities

**Published:** 1876-11

**Authors:** 


### HEALTH OF CITIES.

The health of large cities is becoming to be regarded more and more as a subject of highest importance. There is a great difference between them; in some cities one person out of every 25 dies annually, while in others there is only one death to every 50 persons. The following table exhibits several cities, in comparison, both European and American, showing in each how many inhabitants are lost by death every year:

| | | | | | |
|---|---|---|---|---|---|
| Portland . . | 1 in 62 | New York . . | 1 in 37 | Nice and | |
| Philadelphia | 1 in 45 | St. Petersburg. | 1 in 37 | Palermo . | 1 in 31 |
| Glasgow . . | 1 in 44 | Charleston. . | 1 in 36 | Madrid . . . | 1 in 29 |
| Manchester | 1 in 44 | Baltimore . . | 1 in 35 | Naples . . . | 1 in 28 |
| Geneva. . . | 1 in 43 | Leghorn . . | 1 in 35 | Brussels . . | 1 in 26 |
| Boston . . . | 1 in 41 | Berlin . . . . | 1 in 34 | Rome . . . | 1 in 25 |
| London. . . | 1 in 40 | Paris&Lyons. | 1 in 32 | | |

*AN IRON CONSTITUTION.*

Is an appropriate figure of speech, as applied to a person of robust organization; for without a sufficiency of iron in the system it can neither be strong nor enduring. Bearing this fact in mind let all who suffer from nervous diseases, or physical debility, whether general or local, put their trust in STAFFORD'S IRON AND SULPHUR POWDERS. The combination is charged with the two elements which science declares that the weak and nervous need—iron, to augment the vital forces, and sulphur, to disinfect the blood and the secretions. For debility, in all its varieties, and whether arising from general or specific and peculiar causes, the Powders are the most potent of all remedies. They are especially adapted to the cure of sexual disabilities.

Sold by Druggists. 1 Package, 12 Powders, $1; 6 Packages, 72 Powders, $5. Mailed Free. HALL & RUCKEL, 218 Greenwich Street, New York.

The demand for Dr. Hall's "Spirometer" has so far exceeded our expectations that we were unable to deliver them promptly. We have now made arrangements that will enable us to fill our orders at once. Price of medium size $5.50; large size, $7.50. Sent free on receipt of price. Address, "Hall's Journal of Health," New York.

